# Physical Activity Level, Barriers, and Facilitators for Exercise Engagement for Chronic Community-Dwelling Stroke Survivors in Low-Income Settings: A Cross-Sectional Study in Benin

**DOI:** 10.3390/ijerph20031784

**Published:** 2023-01-18

**Authors:** Sènadé Inès Noukpo, Lisa Tedesco Triccas, Bruno Bonnechère, Thierry Adoukonou, Peter Feys, Oyéné Kossi

**Affiliations:** 1 Unit of Neurology and NeuroRehabilitation, University Hospital of Parakou, Parakou 01 BP 02, Benin; 2 REVAL, Rehabilitation Research Center, Hasselt University, 3590 Diepenbeek, Belgium; 3 Technology-Supported and Data-Driven Rehabilitation, Data Science Institute, Hasselt University, 3590 Diepenbeek, Belgium; 4 ENATSE, National School of Public Health and Epidemiology, University of Parakou, Parakou 03 BP 10, Benin; 5 Department of Neurology, Faculty of Medicine, University of Parakou, Parakou 03 BP 10, Benin

**Keywords:** exercise barriers, exercise preferences, physical activity, stroke

## Abstract

After a stroke incident, physical inactivity is common. People with stroke may perceive several barriers to performing physical activity (PA). This study aimed to document the PA level and understand the barriers and facilitators to engaging in PA for community-dwelling stroke survivors in Benin, a lower middle-income country. A cross-sectional study was conducted in three hospitals in Benin. Levels of PA were recorded by means of the Benin version of the International Physical Activity Questionnaire long form (IPAQ-LF-Benin), which is validated for stroke survivors in Benin. The perceived exercise facilitators and barriers were assessed by the Stroke Exercise Preference Inventory-13 (SEPI-13). A descriptive analysis and associations were performed with a Confidence Interval of 95% and <0.05 level of significance. A total of 87 participants (52 men, mean age of 53 ± 10 years, mean time after a stroke of 11 (IQR: 15) months and an average of 264.5 ± 178.9 m as distance on the 6 min walking test (6MWT) were included. Overall, stroke survivors in Benin reached a total PA of 985.5 (IQR: 2520) metabolic equivalent (METs)-minutes per week and were least active at work, domestic, and leisure domains with 0 MET-minutes per week. The overview of PA level showed that 52.9% of participants performed low PA intensity. However, 41.4% performed moderate PA or walking per day for at least five days per week. Important perceived barriers were lack of information (45.3%), hard-to-start exercise (39.5%), and travelling to places to exercise (29.9%). The preference for exercise was with family or friends, outdoors, for relaxation or enjoyment (90.2%), and receiving feedback (78.3%). Several socio-demographic, clinical, and community factors were significantly associated with moderate or intense PA (*p* < 0.05) in stroke survivors in this study. Our findings show that the PA level among chronic stroke survivors in Benin is overall too low relative to their walking capacity. Cultural factors in terms of the overprotection of the patients by their entourage and/or the low health literacy of populations to understand the effect of PA on their health may play a role. There is a need for new approaches that consider the individual barriers and facilitators to exercise.

## 1. Introduction

Stroke is a neurological disorder with a vascular origin involving an acute focal injury of the central nervous system [1]. According to the Global Burden of Disease, Injuries and Risk Factors Study (2019), stroke is the second-leading cause of death worldwide. Specifically, in 2019, in lower-middle-income countries such as Benin, the age-standardized stroke prevalence was 1100–1300 per 100,000 persons [2,3]. Compared to the age-standardized stroke prevalence of many western European countries, the prevalence in Benin is almost double [4]. Stroke survivors often have functional limitations, which lead to reduced physical activity (PA) and deconditioning associated with deterioration in quality of life [5,6]. Regular participation in PA can prevent the recurrence of stroke and improve walking capacity, muscle strength, bone density, functional mobility, and also quality of life [7,8,9]. Some studies have examined sedentary behavior in community-dwelling stroke survivors [10,11,12,13]: for example, Moore et al. (2015) found that within seven days of stroke in the community, individuals took 63% fewer steps per day than healthy individuals and were sedentary for 23 h per day [14].

Several randomized controlled trials have demonstrated the benefits of PA after stroke in increasing physical function and improving fitness [15,16,17,18]. Recently, systematic reviews, including updated Cochrane reviews, showed that cardiorespiratory training, including walking, reduces disability and dependence on others during ambulation and improves walking speed in stroke survivors [9,19]. Consequently, PA is recommended for stroke survivors in several national clinical guidelines, including guidelines from the American Heart Association and Scottish Intercollegiate Guidelines Network [7,20]. The promotion of PA in stroke survivors must emphasize 150 min per week of moderate-intensity aerobic activity and muscle-strengthening activity; to reduce sedentary behavior and risk management for secondary prevention of stroke [7,21]. Despite these recommendations, around 70% of stroke survivors undertake minimal post-rehabilitation PA [22], causing low fitness levels compared to age-matched peers.

Studies mostly from developed countries reported varied barriers such as poor health, lack of company, physical impairments from stroke, lack of motivation, and environmental factors. However, facilitators such as motivation, social support, and planned activities to fill empty schedules for PA among stroke survivors were reported [23,24,25]. Identifying the exercise barriers and facilitators for stroke survivors in low-income settings may help to improve engagement in PA by diminishing the perceived barriers and to promote the facilitators [26,27]. To the best of our knowledge, there is no single study exclusively reporting barriers and facilitators to PA among stroke survivors in Benin; there also seems to be limited published studies on perceived barriers reported by stroke survivors generally.

The main aim of this cross-sectional study was to investigate the level of PA and understand barriers and facilitators to engaging in physical activity for chronic community-dwelling stroke survivors in Benin, a lower middle-income country. An additional objective was to assess the participants’ factors that could influence the level of PA in people post-stroke.

## 2. Materials and Methods

### 2.1. Study Design and Ethics Considerations

A descriptive cross-sectional design with a systematic recruitment sampling technique was conducted in this study. The study was approved by the Ethics Committee of biomedical research of the University of Parakou [REF 0490/CLERB-UP/P/SP/R/SA, 4 October 2021]. The objectives and methodology of the study were explained to the participants prior to their participation, and written informed consent was obtained from all participants.

### 2.2. Setting

The study was conducted in three hospitals in Benin: the University Hospital of Parakou, the Hospital of Abomey, and the University Hospital of Porto Novo, from 10 October 2021 to 15 February 2022.

### 2.3. Participants and Recruitment

A systematic recruiting approach was used, and potential participants were identified through the admission records from the three aforementioned hospitals. To be included, participants had to: (1) present a unilateral stroke in the chronic stage (at least 3 months after onset), (2) be an adult (≥18 years), (3) have absence or minimal disability with a modified Ranking Scale (mRS) score ≤ 3 [28], (4) have absence of cognitive impairment (Community Screening Instrument for Dementia (CSI-D) score ≥ 7) [29], and (5) be able to give consent. The exclusion criterion was a contraindication to PA, such as asthma and heart failure. A total of 87 patients met the inclusion criteria over the recruitment period and were voluntarily involved in the study.

### 2.4. Procedure

Participants were invited to the hospitals where the clinical data were collected, and the questionnaires were completed. Firstly, the participants’ characteristics were collected. These included the socio-demographic data (age, gender, marital status, professional status, instruction level, social insurance), clinical data (stroke type, stroke topography, affected hemisphere, stroke risk factor, time post-stroke, time follow-up physiotherapy, other therapy), and community environment data (living condition, access to home, life situation, driving status, support available). Functional data consisting of the severity of disability assessed by modified Rankin Scale (mRS) [28], Functional Ambulatory Classification (FAC) [29], walking distance and walking endurance evaluated with Six Minute Walking Test (6MWT) [30], Borg scale (perceived fatigue after effort) [31] and Time Up and Go test (walking and balance) [32]. Secondly, the main outcome measures, the International Physical Activity Questionnaire long form Benin (IPAQ-LF-Benin) [33] and the Stroke Exercise Preference Inventory-13 (SEPI-13) [34], were administered. All data were collected by three trained physiotherapists in these different hospitals.

### 2.5. Outcome Measures

#### 2.5.1. International Physical Activity Questionnaire-Long Form (IPAQ-LF-Benin)

IPAQ-LF-Benin is a Benin version of the IPAQ-LF questionnaire. This is a valid and reliable questionnaire for measuring the level of PA in people post-stroke living in the community [35]. The original long-form questionnaire was adapted and validated in healthy people and stroke survivors in the Beninese cultural context in 2019. PA is assessed in four domains, including work, transport, domestic and gardening, and leisure time. The IPAQ-LF-Benin showed excellent test–retest reliability of the different domains and the sedentary time (all ICCs ≥ 0.94) [36]. In addition, the questionnaire collects specific information about walking, moderate-intensive, and vigorous-intensive PA in each of these four domains. The participants were also asked to document the volume of their PA. This volume was computed by weighting each type of activity by its energy requirements defined in metabolic equivalent (METs) [36]. In this way, a score in MET-minutes was given for each domain, each intensity category, and the total PA. Afterward, participants were classified into three levels of PA, namely low, moderate, and high [36].

#### 2.5.2. Stroke Exercise Preference Inventory-13 (SEPI-13)

SEPI-13 is a questionnaire that assesses the exercise preferences (facilitators) and barriers of stroke survivors [37]. The questionnaire contains 13 items providing a parsimonious measure for assessing key exercise preferences in the stroke population and a 9-item module on barriers to exercise participation after stroke. For each item, the participants chose a number between 0% (don’t agree at all) and 100% (totally agree) to indicate their level of agreement [37]. The scoring template consists of 7 exercise preference factors: (1) Supervision-support, (2) Confidence-challenge, (3) Health-wellbeing, (4) Similar others, (5) Exercise context, (6) Home-alone, and (7) Music-TV; to calculate average factor scores on factors 1–6 (factor 7 is a single item score). Means and standard deviations for each factor are provided as a reference (taken from a sample of 134 chronic stroke survivors).

### 2.6. Statistical Analysis

Statistical analyses were conducted using SPSS version 25.0. For descriptive purposes, medians and interquartile ranges or means ± standard deviations were calculated for continuous variables and absolute numbers and percentages for categorical variables. The physical activity scores assessed with IPAQ were not normally distributed, so they were transformed into ordinal variables, and ordinal logistic regression was used. The quantitative variables involved were transformed into categorical variables. So to analyze the relationships between participant characteristics and the PA level with expected values higher than five, the Pearson Chi-square test was used. If the expected values were less than five, a Fisher’s exact test was performed. To estimate the association between the main variable and covariates, odds ratios and their CI at 95% were performed for the categories. The level of significance was set to 0.05 throughout the analysis.

## 3. Results

### 3.1. Participants

A total of 572 community-dwelling stroke survivors were identified to participate in this study. Participants were distributed in Parakou (*n* = 218), Abomey (*n* = 189), and Porto Novo (*n* = 165), and after checking for eligibility, 87 participants were included. The selection and inclusion of participants are presented in the flow chart in Supplementary Materials Figure S1.

### 3.2. Description of the Sample

The 87 included participants were 53 ± 10 years of age (range 26–74), and 52 of them were men. Among the participants, 83.9% were married and did not have social insurance (56.3%). Almost thirty had a secondary education level (34.5%) and were employed (35.6%)**.** All participants had a stroke at least 3 months before the inclusion, with a mean time after stroke of 11 (IQR: 15) months. From the total, 71.3% experienced an ischemic stroke, 36.8% had high blood pressure, and 43.6% had at least two vascular risk factors. Relating to the functional data, the majority of participants (60%) were mildly disabled (score mRS ≤ 3), and 72% had good ambulatory functional capacity (FAC ≥ 4). The average distance on the 6MWT was 264.45 ± 178.9 m. There was no significant difference (*p* = 0.64) when comparing the average distance covered at the first minute (44.18 m ± 24.6) and at the sixth minute (42.39 m ± 24.0), indicating that no motor fatigability was present. On the Borg scale, only 2% of the participants perceived a maximum effort (Borg [17,18,19,20]), 29% perceived a hard effort (Borg [12,13,14,15,16]), and 69% perceived a light effort (Borg [6,7,8,9,10,11]) immediately after this walking test. Regarding home and community environment characteristics, 97.7% of the participants lived on the ground floor, and 71.3% had dependent children living in their houses. Most had family to support them (85.1%), 37.9% were dependent on caregivers, and 23% took public transportation. The description of the sample is summarized in the second column of Table 1.

### 3.3. Physical Activity Level and Perceived Barriers and Facilitators for Stroke Survivors

#### 3.3.1. Level of PA Practice for People with Stroke

The PA levels of the stroke patients, measured with IPAQ-LF-Benin, is shown in Table 2. People with stroke reached 985.5 (IQR: 2520) MET-minutes per week as total PA level. Looking at the specific domains of the IPAQ-LF-Benin, participants were mainly active in the transport domain with a median MET-minutes per week of 231 (IQR: 693). Participants were least active at work, domestic, and leisure domains, with a median MET-minutes per week of 0. Thus it was seen that more than 75% of the subjects obtained a zero PA intensity score in all these three IPAQ domains and that only 25% of the subjects had a score of 370 MET-minutes per week and 400 MET-minutes per week, respectively, in the domestic and leisure domains. Although some had a score in three domains of IPAQ (transportation, domestic, and leisure), they did not reach the 600 MET-minutes per week required to qualify them as moderately sufficient activity except in transport, where 25% reached 693 MET-minutes per week. This means that at least half of the patients did not perform moderate to intense physical activities. Note that in the area of work where the central tendencies gave 0, there were some who gave results (see the maximum in Table 1).

Regarding the three sub-categories of each domain of the IPAQ, we found the median MET-minutes per week of the walking activity category had the highest median MET-minutes per week with a value of 396 (IQR: 1254). The participants performed the least activity in the vigorous activity category with a median MET-minutes per week of 0. This means that at least half of the sample did not do any vigorous walking activity.

Summarizing the participants’ total PA intensity into three levels (low, moderate, and high) as shown in Table 3, few (5.8%) of the sample performed intense PA at least three days per week, resulting in energy expenditure of at least 1500 METs, unlike 52.9% who had low PA intensity. Nearly half of the participants (41.4%) performed moderate PA or walking per day for at least five days per week.

The cross-analysis of the level of PA of the participants by IPAQ domains showed that of the 21 subjects who had work activity, half (55.3%) practiced moderate PA. For the active transport domain, it is walking that predominated (92.0%) in 62 stroke survivors. In the fields of domestic and gardening activities, moderate yard chores were most frequently done (56.4%), and walking was rated as the highest in the leisure domain (62.9%), respectively, in 35 and 41 participants. Individuals spent an average time of 5 days per week for at least 30 min per session on predominant PA domains. More details can be found in Supplementary Materials Tables S1 and S2.

#### 3.3.2. Barriers and Facilitators

**Figure 1:**
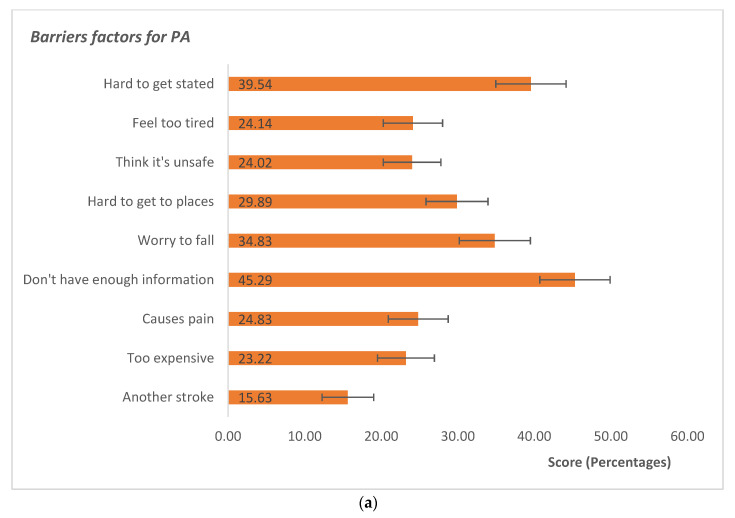

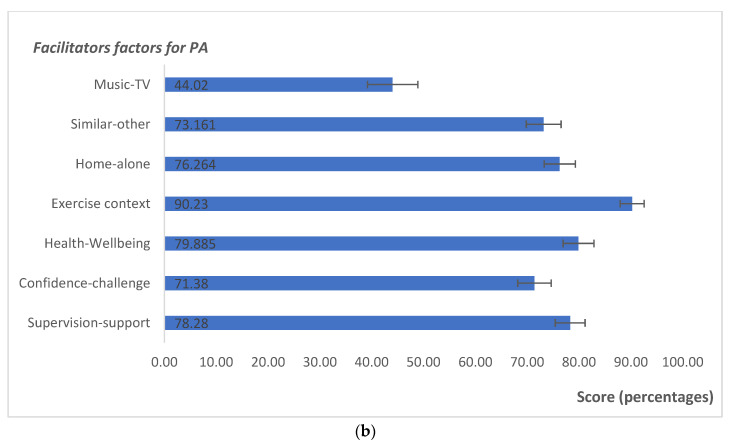
Participants perceived barriers (**a**) and facilitators (**b**) to engage in physical activity measured by SEPI-13 (percentage mean with confidence interval).

Figure 1 shows the Barriers and facilitators factors of PA in our sample measured with SEPI-13. Beninese participants with stroke reported a lack of information about exercise as the highest barrier, 45.3% of the score. Furthermore, hard getting started to exercise (score = 39.5%), fear of falling (score = 34.8%), and hard getting to places to exercise (score = 29.9%) were reported as the three highest barriers. The participants (score = 15.6%) were worried that exercise might cause them to have another stroke. Regarding the facilitators, the main factor was “exercise context” with 90.2% of responses, which means a preference for exercise with family or friends, outdoors, for relaxation or enjoyment. Other important facilitators were the “health-wellbeing” and “supervision-support” factors with, respectively, a mean response of 79.9% and 78.3% indicating that a major part of the participants liked to exercise for health that makes them feel good. In addition, they preferred a trained instructor for receiving feedback and support. The lowest facilitator was the “music-TV” factor, with a mean response percentage of 44.0; thereby, listening to music or watching TV during exercise was less important for the participants.

### 3.4. Relationship between PA Level and Other Factors

Participants’ characteristics associated with the level of PA are shown in Table 3. The socio-demographic characteristics, such as marital status and social insurance, were significantly associated with moderate to intense PA with, respectively, a *p*-value of 0.001 and 0.015. Regarding the clinical data, stroke topography (*p* = 0.036), vascular risk factor before stroke (*p* = 0.021), level of disability (*p* = 0.014), functional ambulatory classification (*p* = 0.019), and effort perception after 6MWT (*p* = 0.049) were significantly related with the level of PA. Community environment characteristics regarding driving status (*p* = 0.038) showed significant associations with the level of PA.

Summarized, the parameters: being married, not having social insurance, having a stroke risk factor (at least 2), having more disability, having fewer ambulatory capacities, and reporting a high perception of effort and dependency on someone for transport, may contribute to explain a low level of physical activity for stroke survivors in Benin.

## 4. Discussion

This study aimed to investigate the level of PA, the perceived barriers and facilitators, and the factors influencing PA level to engage in PA in chronic community-dwelling stroke survivors in Benin. Participants were mainly active in the transport domain and were least active in the work, domestic, and leisure domains. Walking activity was the highest physical activity, and more than half of stroke survivors had a low level of PA.

To stimulate health benefits, it is recommended a minimum of 150 min per week of moderate-intensity PA (30 mn per day for at least 5 days) [21]. Only 41.4% of participants reached this recommendation, 5.7% reached the high level, and 52.9% had a low level which meant a low level of AP for stroke survivors in Benin. A similar result was observed by Houehanou et al. (2022) [38] in an urban community of Parakou, Northern Benin, with (a 51.1%) prevalence of low physical activity practice for stroke survivors. Looking at the study of Idowu et al. (2015), 80.2% of the Nigerian chronic stroke participants had a low PA level evaluated with the IPAQ-LF [26]. In addition, given that Nigeria is a neighboring country of Benin and also a lower-middle-income country, the results can be compared to some extent. These results show an inconsistency regarding the PA level of chronic stroke participants. Current literature reports that the prevalence of low PA among chronic stroke survivors in high-income countries ranges from 45.4 to 53.5%, within which falls the prevalence found in the present study [39,40]. In this study, men were less active than women in the leisure domain and were most active in the work domain. This can be explained by the fact that usually, in African countries, women take care of domestic activities, and men work outdoors [41,42]. Prior research has also shown that individuals in low-income countries are less likely to engage in leisure time activities compared with high income countries [43].

The results of this study showed that people with chronic stroke in Benin prefer to exercise with family or friends, outdoors, for relaxation or enjoyment. This may explain why there is a significant association between the fact of having children and the practice of PA. In addition, a major proportion of the participants liked to exercise for health, which makes them feel good, as part of their daily activities. Moreover, they preferred a trained instructor, receiving feedback, and having someone at hand to help if needed; but listening to music or watching TV during exercise was less important. There were some similarities and some differences in perceived barriers to PA, while facilitators to PA resonated with the literature. Our result was similar to the current literature showing that facilitators related to physical health were the most common [37,44]. Barriers identified in our study were mainly a lack of information about exercise, fear of falling, getting started to exercise, and getting to places to exercise. This is in line with the two reviews that reported barriers and facilitators for PA participation in disabled individuals [45,46]. Since the study was conducted in a low-middle-income country where facilities such as stroke groups or community rehabilitation centers are rare [47], participants did not have the opportunity to participate in adaptive PA every day. Similar results were found in the current literature in low-, middle-, and high-income countries [26,44,48]. Therefore, it is important to provide sufficient information, accessibility, and safety in the development of an exercise program with a focus on health and wellbeing for people with chronic stroke.

The results of our study also show that individuals living in Benin perceived different barriers to PA compared with those documented in the literature. For example, in a systematic review by Nicholson et al. (2012), the most commonly reported barriers were environmental issues (access, transportation, and cost) [49]. However, Benin stroke survivors did not perceive environmental barriers to be among the obstacles to accessing PA. The most commonly reported barrier to PA in this study (i.e., fear of falling) was also reported by stroke survivors living in England (*n* = 76.70% > 1 year post-stroke) [50]. In addition, variability in the exercise facilitators and barriers is seen by high standard deviations. This was also found in the literature [37]. Therefore, a stroke exercise program should be made individually and should take into account personal facilitators and barriers.

Being married, not having social insurance, having a stroke risk factor (at least 2), having more disability, having a less ambulatory condition, having a high perception of effort, and being dependent on someone for transport were factors associated with a moderate to intense PA. Our results were in agreement with some of the literature [49,51,52], which found that a major barrier to following the treatment plan and/or a healthy lifestyle including PA was income and/or lack of insurance [52]. Webb and Gonzalez (2006) [53] noted that communication between the healthcare provider and the client might be enhanced by the healthcare provider becoming more aware of issues that affect the client, such as financial stress.

As having a vascular risk factor may inhibit PA in stroke survivors, some authors also found that stroke survivors with hypertension were less likely to engage in recommended aerobic activity [54,55,56]. In addition, it was found in the literature that those who were married and lived with their spouse were less likely to have a long period of sedentary life and therefore a better level of PA [57,58], which is in line with the findings of the present study, which showed that most participants like to exercise with family or friends.

### 4.1. Strengths and Limitations

The use of IPAQ-Bénin is a real asset because it is validated according to the culture and the French spoken in Benin and is, therefore, the best instrument for measuring the physical activity of stroke survivors in Benin. In addition, the SEPI-13 ensured that we investigated a wide range of personal and environmental contextual influences and were successful in categorizing participants’ responses to meaningful theoretical domains. Participants were all at least three months post-stroke and had been discharged from hospital. This allowed us to understand the difficulties these participants faced after discharge and throughout their community-dwelling stay post-stroke. Another strength of this study was that the level of PA and the exercise facilitators and barriers are broadly inventoried among Beninese chronic stroke survivors. This inventory can provide the information to adequately set up a stroke rehabilitation program.

An interview bias by the participant was possibly present when taking the IPAQ-LF-Benin by giving socially desirable answers [59]. This is the case of sitting time questions with the IPAQ-LF-Benin that demonstrated invalid results. Thereby, the results of the sitting time were not included in the statistical analysis. Considering all these remarks, the results of the IPAQ-LF-Benin and SEPI-13 may be biased.

### 4.2. Implications and Recommendations for Clinical Field and Future Research

The results of this study showed the need to increase the PA level among chronic stroke survivors in Benin. There is a need for additional approaches that consider the individual exercise facilitators and barriers to improving the level of PA in people with chronic stroke. Home-based rehabilitation programs already showed positive results in improving the level of PA in this population [60]. Additionally, PA after stroke can be increased by using mobile health (mHealth) applications [61]. Thereby, home-based or mHealth rehabilitation programs are potential additional approaches to stimulate the level of PA in chronic stroke survivors. Moreover, these approaches aiming at PA are easy and at a low cost and therefore applicable in low- and middle-income countries [62]. In this way, considering the exercise facilitators and barriers in these additional rehabilitation programs could be a successful solution to increase PA in chronic stroke survivors.

## 5. Conclusions

The results of this study showed that most of the chronic community-dwelling stroke survivors in Benin did not meet PA recommendations. Additionally, there is a need for new approaches that consider the individual exercise facilitators and barriers to improving the level of PA in people with chronic stroke. Services must adapt to address these perceived barriers and build upon the perceived facilitators. These data have implications for health care and exercise professionals who wish to help stroke survivors to become more physically active by allowing targeted interventions to be designed and delivered.

## Figures and Tables

**Table 1:** Stroke survivors’ characteristics associated with moderate-intense PA.

	Sample Mean ± SD/Median (IQR)/*n* (%)	PA Moderate-Intense		OR (95% IC)	*p*
		Yes	No		
Socio demographic factors					
Age (years)	53.6 ± 10.3	-	-		
Age category (years)					
<50	29 (33.3)	19 (65.5)	10 (34.5)	1	
50–60	32 (36.8)	18 (56.3)	14 (43.7)	1.5 [0.5–4.2]	0.459
>60	26 (29.9)	13 (50.0)	13 (50.0)	1.9 [0.6–5.6]	0.244
Sex					
Male	52 (59.8)	29 (55.8)	23 (44.2)	1	
Female	35 (40.2)	20 (57.1)	15 (42.9)	0.9 [0.4–2.2]	0.899
Marital status					
Married	73 (83.9)	36 (49.3)	37 (50.7)	1	
Single/Divorced/widower	14 (16.1)	13 (92.9)	1 (7.1)	0.07 [0.01–0.6]	0.001 *
Profession					
Employed	31 (35.6)	21 (67.8)	10 (32.2)	1	
Independent	23 (26.4)	11 (47.8)	12 (52.2)	2.3 [0.7–6.9]	0.140
Unemployed/household/other	33 (37.9)	17 (51.5)	16 (48.5)	1.9 [0.7–5.5]	0.186
Education level					
Illiterate	11 (12.6)	6 (57.6)	5 (42.4)	1	
Primary	28 (32.5)	13 (46.4)	15 (53.6)	1.4 [0.3–5.6]	0.648
Secondary	30 (34.5)	17 (56.7)	13 (43.3)	0.9 [0.2–3.7]	0.903
Superior	18 (20.7)	13 (72.2)	5 (27.8)	0.4 [0.09–2.2]	0.331
Social insurance					
Yes	38 (43.7)	11 (28.9)	27 (71.1)	3.0 [1.2–7.4]	0.015 *
No	49 (56.3)	27 (55.1)	22 (44.9)	1	
Clinical characteristics					
Type of stroke					
Ischemic	62 (71.3)	36 (58.1)	26 (41.9)	1	
Hemorrhagic	18 (20.7)	7 (38.9)	11 (61.1)	2.2 [0.7–6.4]	0.151
Non determined	7 (8.0)	6 (85.7)	1 (14.3)	0.2 [0.03–2.0]	0.156
Time after stroke (months)	11 (IQR: 15)	-	-		
Time after stroke category (months)					
[0–6]	25 (28.7)	16 (64.0)	9 (36.0)	1	
[6–12]	25 (28.7)	17 (68.0)	8 (32.0)	0.8 [0.3–2.7]	0.765
>12	37 (42.6)	16 (43.2)	21 (56.8)	2.3 [0.8–6.6]	0.108
Vascular risk factors (before stroke)					
None	13 (14.9)	2 (15.4)	11 (84.6)	6.8 [1.3–34.9]	0.021 *
High blood pressure	32 (36.8)	11 (34.4)	21 (65.6)	2.3 [0.9–6.2]	0.081
Diabetes	4 (4.6)	4 (100.0)	0 (0.0)	-	
At least two risk factors/other	38 (43.6)	21 (55.3)	17 (44.7)	1	
Stroke topography					
Unknown	35 (40.2)	19 (54.3)	16 (45.7)	1	
Mild cerebral artery	32 (36.8)	22 (68.8)	10 (31.2)	0.5 [0.1–1.5]	0.224
Cerebral anterior/Cerebral posterior	6 (6.9)	5 (83.3)	1 (16.7)	0.2 [0.02–2.2]	0.190
Other	14 (16.1)	3 (21.4)	11 (78.6)	4.4 [1.0–18.4]]	0.036 *
Affected hemisphere					
Left	56 (64.4)	23 (41.1)	33 (58.9)	1	
Right	31 (35.6)	15 (48.4)	16 (51.6)	0.7 [0.3–1.8]	0.510
Functional rehabilitation after stroke (months)					
<1	28 (32.2)	8 (28.6)	20 (71.4)	2.2 [0.7–6.5]	0.156
1–3	30 (34.5)	14 (46.7)	16 (53.3)	1	
4–6	13 (14.9)	8 (61.5)	5 (38.5)	0.5 [0.1–2.1]	0.370
>6	16 (18.4)	8 (50.0)	8 (50.0)	0.9 [0.3–2.9]	0.829
Other therapy					
No	19 (21.8)	10 (52.6)	9 (47.4)	1.2 [0.4–3.4]	0.714
Yes	68 (78.2)	39 (57.4)	29 (42.6)	1	
Modified Rankin Scale					
<3	60 (69.0)	21 (35.0)	39 (65.0)	1	
≥3	27 (31.0)	17 (63.0)	10 (37.0)	0.3 [0.1–0.8]	0.014 *
Functional Ambulatory Classification					
<4	15 (17.2)	11 (73.3)	4 (26.7)	0.2 [0.1–0.8]	0.019 *
≥4	72 (82.7)	27 (37.5)	45 (62.5)	1	
6MWT (m)	245.0 ± 178.9	-	-		
Borg (after 6MWT)					
≥12	27 (31.0)	16 (59.3)	11 (40.7)	0.4 [0.2–1.0]	0.049 *
<12	60 (69.0)	22 (36.7)	38 (63.3)	1	
Home and community factors					
Living condition					
Family home	29 (33.3)	16 (55.2)	13 (44.8)	1.0 [0.4–2.4]	0.968
Apartment	5 (5.7)	4 (80.0)	1 (20.0)	0.3 [0.03–2.9]	0.275
House home	53 (60.9)	29 (54.7)	24 (45.3)	1	
Living situation					
Living alone	4 (4.6)	3 (75.0)	1 (25.0)	0.6 [0.1–5.8]	0.537
Dependent children	62 (71.3)	39 (62.9)	23 (37.1)	1	
Living with others	21 (24.1)	14 (66.7)	7 (33.3)	0.8 [0.3–2.4]	0.756
Driving status					
Independent	34 (49.1)	22 (64.7)	12 (35.3)	1	
Dependent on caregiver	33 (37.9)	13 (39.4)	20 (60.6)	2.8 [1.0–7.6]	0.038 *
Public transportation	20 (23.0)	14 (70.0)	6 (30.0)	0.8 [0.2–2.6]	0.690
Available support					
No	13 (14.9)	9 (69.2)	4 (30.8)	0.5 [0.1–1.8]	0.375
Yes	74 (85.1)	40 (54.1)	34 (45.9)	1	

* Statistically significant (*p* < 0.05).

**Table 2:** Results of the IPAQ-LF-Benin.

Intensity of Activity	Median MET-Minutes/Week (IQR)	Minimum	Maximum
Total physical activity	985.5 (IQR:2520)	0	56,826
MET-minutes/week per domain			
Total work	0 (IQR: 0)	0	20,853
Total transport	231 (IQR:693)	0	5544
Total domestic	0 (IQR:370)	0	35,280
Total leisure	0 (IQR:400)	0	6344
MET-minutes/week per domain category			
Total walking activity	396 (IQR:1254)	0	12,078
Total moderate activity	120 (IQR:540)	0	55,440
Total vigorous activity	0 (IQR: 0)	0	7200

**Table 3:** PA intensity resulting in energy expenditure.

Type of Physical Activity		Sex		Age Category		
		Male (*n* = 52)	Female (*n* = 35)	[20–40] (n = 6)	[40–60] (n = 58)	[60–80] (n = 23)
Intense PA at least 3 days/week, resulting in energy expenditure of at least 1500, *N* (%)	No	50 (57.5)	32 (36.8)	6 (6.9)	55 (63.2)	21 (24.1)
	Yes	2 (2.3)	3 (3.4)	0 (0.0)	3 (3.5)	2 (2.3)
At least 30 min of moderate PA or walking/day for 5 days or more/week, *N* (%)	No	26 (29.9)	25 (28.7)	3 (3.5)	33 (37.9)	15 (17.2)
	Yes	26 (29.9)	10 (11.5)	3 (3.5)	25 (28.7)	8 (9.2)
Low intensity, *N* (%)	No	26 (29.9)	13 (14.9)	5 (5.7)	22(25.3)	12 (13.8)
	Yes	26 (29.9)	22 (25.3)	3 (3.5)	31 (35.6)	14 (16.1)
Level of activity			*N* (%)			
Low			46 (52.9%)			
Moderate			36 (41.4%)			
High			5 (5.7%)			

## Data Availability

Not applicable.

## Supplementary Materials

The following supporting information can be downloaded at: https://www.mdpi.com/article/10.3390/ijerph20031784/s1.

## References

[1] Sacco R.L., Kasner S.E., Broderick J.P., Caplan L.R., Connors J., Culebras A., Elkind M.S., George M.G., Hamdan A.D., Higashida R.T. (2013). An updated definition of stroke for the 21st century: A statement for healthcare professionals from the American Heart Association/American Stroke Association. Stroke.

[2] Adoukonou T., Kossi O., Fotso Mefo P., Agbetou M., Magne J., Gbaguidi G., Houinato D., Preux P.-M., Lacroix P. (2021). Stroke case fatality in sub-Saharan Africa: Systematic review and meta-analysis. Int. J. Stroke.

[3] Adoukonou T., Kossi O., Agbétou M., Tchaou B., Agballa G., Houinato D. (2018). Short term (3 months) prognosis of stroke in Parakou. Neurosci. Med..

[4] Roth G.A., Mensah G.A., Johnson C.O., Addolorato G., Ammirati E., Baddour L.M., Barengo N.C., Beaton A.Z., Benjamin E.J., Benziger C.P. (2020). Global burden of cardiovascular diseases and risk factors, 1990–2019: Update from the GBD 2019 study. J. Am. Coll. Cardiol..

[5] Rimmer J.H., Wang E., Smith D. (2008). Barriers associated with exercise and community access for individuals with stroke. J. Rehabil. Res. Dev..

[6] Noukpo S.I., Kossi O., Triccas L.T., Adoukonou T., Feys P. (2022). Content and effectiveness of community-based rehabilitation on quality of life in people post stroke: A systematic review with meta-analysis. Disabil. CBR Incl. Dev..

[7] Billinger S.A., Arena R., Bernhardt J., Eng J.J., Franklin B.A., Johnson C.M., Mackay-Lyons M., Macko R.F., Mead G.E., Roth E.J. (2014). Physical activity and exercise recommendations for stroke survivors: A statement for healthcare professionals from the American Heart Association/American Stroke Association. Stroke.

[8] Morris J.H., Macgillivray S., McFarlane S. (2014). Interventions to promote long-term participation in physical activity after stroke: A systematic review of the literature. Arch. Phys. Med. Rehabil..

[9] Nindorera F., Nduwimana I., Thonnard J.L., Kossi O. (2021). Effectiveness of walking training on balance, motor functions, activity, participation and quality of life in people with chronic stroke: A systematic review with meta-analysis and meta-regression of recent randomized controlled trials. Disabil. Rehabil..

[10] Ainsworth B.E., Haskell W.L., Herrmann S.D., Meckes N., Bassett D.R., Tudor-Locke C., Greer J.L., Vezina J., Whitt-Glover M.C., Leon A.S. (2011). 2011 Compendium of Physical Activities: A second update of codes and MET values. Med. Sci. Sports Exerc..

[11] Tremblay M. (2012). Letter to the Editor: Standardised use of the terms “sedentary” and “sedentary behaviours". Afr. J. Phys. Act. Health Sci..

[12] Gill J.M., Bhopal R., Douglas A., Wallia S., Bhopal R., Sheikh A., Forbes J.F., McKnight J., Sattar N., Murray G. (2011). Sitting time and waist circumference are associated with glycemia in UK South Asians: Data from 1,228 adults screened for the PODOSA trial. Diabetes Care.

[13] Veerman J.L., Healy G.N., Cobiac L.J., Vos T., Winkler E.A., Owen N., Dunstan D.W. (2012). Television viewing time and reduced life expectancy: A life table analysis. Br. J. Sports Med..

[14] Moore S.A., Hallsworth K., Jakovljevic D.G., Blamire A.M., He J., Ford G.A., Rochester L., Trenell M.I. (2015). Effects of community exercise therapy on metabolic, brain, physical, and cognitive function following stroke: A randomized controlled pilot trial. Neurorehabil. Neural Repair.

[15] Cramp M.C., Greenwood R.J., Gill M., Lehmann A., Rothwell J.C., Scott O.M. (2010). Effectiveness of a community-based low intensity exercise programme for ambulatory stroke survivors. Disabil. Rehabil..

[16] Mead G.E., Greig C.A., Cunningham I., Lewis S.J., Dinan S., Saunders D.H., Fitzsimons C., Young A. (2007). Stroke: A randomized trial of exercise or relaxation. J. Am. Geriatr. Soc..

[17] Macko R.F., Ivey F.M., Forrester L.W., Hanley D., Sorkin J.D., Katzel L.I., Silver K.H., Goldberg A.P. (2005). Treadmill exercise rehabilitation improves ambulatory function and cardiovascular fitness in patients with chronic stroke: A randomized, controlled trial. Stroke.

[18] Mudge S., Barber P.A., Stott N.S. (2009). Circuit-based rehabilitation improves gait endurance but not usual walking activity in chronic stroke: A randomized controlled trial. Arch. Phys. Med. Rehabil..

[19] Saunders D.H., Sanderson M., Hayes S., Johnson L., Kramer S., Carter D.D., Jarvis H., Brazzelli M., Mead G.E. (2020). Physical fitness training for stroke patients. Cochrane Database Syst. Rev..

[20] Scottish Intercollegiate Guidelines Network (2008). Management of Patients with Stroke or TIA: Assessment, Investigation, Immediate Management and Secondary Prevention: A National Clinical Guideline.

[21] Kaminsky L.A., Montoye A.H. (2014). Physical activity and health: What is the best dose?. J. Am. Heart Assoc..

[22] Butler E.N., Evenson K.R. (2014). Prevalence of physical activity and sedentary behavior among stroke survivors in the United States. Top. Stroke Rehabil..

[23] Adeniyi A., Idowu O., Ogwumike O., Adeniyi C. (2012). Comparative influence of self-efficacy, social support and perceiived barriers on low physical activity development in patients with type 2 diabetes, hypertension or stroke. Ethiop. J. Health Sci..

[24] McDonnell M.N., Esterman A.J., Williams R.S., Walker J., Mackintosh S.F. (2014). Physical activity habits and preferences in the month prior to a first-ever stroke. PeerJ.

[25] Damush T.M., Plue L., Bakas T., Schmid A., Williams L.S. (2007). Barriers and facilitators to exercise among stroke survivors. Rehabil. Nurs..

[26] Idowu O.A., Adeniyi A.F., Ogwumike O.O., Fawole H.O., Akinrolie O. (2015). Perceived barriers to physical activity among Nigerian stroke survivors. Pan Afr. Med. J..

[27] English C., Manns P.J., Tucak C., Bernhardt J. (2014). Physical activity and sedentary behaviors in people with stroke living in the community: A systematic review. Phys. Ther..

[28] Van Swieten J., Koudstaal P., Visser M., Schouten H., Van Gijn J. (1988). Interobserver agreement for the assessment of handicap in stroke patients. stroke.

[29] Hall K.S., Gao S., Emsley C.L., Ogunniyi A.O., Morgan O., Hendrie H.C. (2000). Community screening interview for dementia (CSI ‘D’); performance in five disparate study sites. Int. J. Geriatr. Psychiatry.

[30] Resnick B., Michael K., Shaughnessy M., Nahm E.S., Kopunek S., Sorkin J., Orwig D., Goldberg A., Macko R.F. (2008). Inflated perceptions of physical activity after stroke: Pairing self-report with physiologic measures. J. Phys. Act. Health.

[31] Chau M.W.R., Chan S.P., Wong Y.W., Lau M.Y.P. (2013). Reliability and validity of the Modified Functional Ambulation Classification in patients with hip fracture. Hong Kong Physiother. J..

[32] Wu G., Sanderson B., Bittner V. (2003). The 6-minute walk test: How important is the learning effect?. Am. Heart J..

[33] Williams N. (2017). The Borg rating of perceived exertion (RPE) scale. Occup. Med..

[34] Wall J.C., Bell C., Campbell S., Davis J. (2000). The Timed Get-up-and-Go test revisited: Measurement of the component tasks. J. Rehabil. Res. Dev..

[35] Honado S.A. (2019). Adaptation et Validation du Questionnaire International de L’activité Physique (IPAQ) Chez Les Personnes Saines et Les Survivants D’un Accident Vasculaire Cérébral au Bénin.

[36] IPAQ Research Committee (2005). International Physical Activity Questionnaire (IPAQ).

[37] Bonner N.S., O’Halloran P.D., Bernhardt J., Cumming T.B. (2016). Developing the stroke exercise preference inventory (SEPI). PLoS ONE.

[38] Houehanou Y.C.N., Agbetou M., Kossi O., Agonnoudé M., Hountada H., Adoukonou T. (2022). Prevalence and factors associated with stroke risk factors in an urban community of Parakou, Northern Benin, 2016. PLoS Glob. Public Health.

[39] Ferreira A.J., Aguiar L.T., Martins J.C., Faria C.D.C.D.M. (2022). Stroke survivors with the same levels of exercise as healthy individuals have lower levels of physical activity. Neurol. Sci..

[40] Ruescas-Nicolau M.-A., Sánchez-Sánchez M.L., Cortés-Amador S., Pérez-Alenda S., Arnal-Gómez A., Climent-Toledo A., Carrasco J.J. (2021). Validity of the international physical activity questionnaire long form for assessing physical activity and sedentary behavior in subjects with chronic stroke. Int. J. Environ. Res. Public. Health.

[41] Adjiwanou V., LeGrand T. (2014). Gender inequality and the use of maternal healthcare services in rural sub-Saharan Africa. Health Place.

[42] Owoo N.S., Lambon-Quayefio M.P. (2021). Mixed methods exploration of Ghanaian women’s domestic work, childcare and effects on their mental health. PLoS ONE.

[43] Teo K., Lear S., Islam S., Mony P., Dehghan M., Li W., Rosengren A., Lopez-Jaramillo P., Diaz R., Oliveira G. (2013). Prevalence of a healthy lifestyle among individuals with cardiovascular disease in high-, middle- and low-income countries: The Prospective Urban Rural Epidemiology (PURE) study. JAMA.

[44] Gagnon M.-A., Batcho C.S., Best K.L. (2022). A description of physical activity behaviors, barriers, and motivators in stroke survivors in Quebec. Disabil. Health J..

[45] Diaz R., Miller E.K., Kraus E., Fredericson M. (2019). Impact of adaptive sports participation on quality of life. Sports Med. Arthrosc. Rev..

[46] Jaarsma E.A., Dijkstra P.U., Geertzen J., Dekker R. (2014). Barriers to and facilitators of sports participation for people with physical disabilities: A systematic review. Scand. J. Med. Sci. Sports.

[47] Pandian J.D., Sudhan P. (2013). Stroke epidemiology and stroke care services in India. J. Stroke.

[48] Débora Pacheco B., Guimaraes Caetano L.C., Amorim Samora G., Sant’Ana R., Fuscaldi Teixeira-Salmela L., Scianni A.A. (2021). Perceived barriers to exercise reported by individuals with stroke, who are able to walk in the community. Disabil. Rehabil..

[49] Nicholson S., Sniehotta F.F., Van Wijck F., Greig C.A., Johnston M., McMurdo M.E., Dennis M., Mead G.E. (2013). A systematic review of perceived barriers and motivators to physical activity after stroke. Int. J. Stroke.

[50] Jackson S.M. (2015). An Investigation of Factors Influencing Physical Activity Levels in People Living in the Community after Stroke.

[51] Simpson L.A., Eng J.J., Tawashy A.E. (2011). Exercise perceptions among people with stroke: Barriers and facilitators to participation. Int. J. Ther. Rehabil..

[52] Ford C.D., Kim M.J., Dancy B.L. (2009). Perceptions of hypertension and contributing personal and environmental factors among rural Southern African American women. Ethn. Dis..

[53] Webb M.S., Gonzalez L.O. (2006). The burden of hypertension: Mental representations of African American women. Issues Ment. Health Nurs..

[54] Choi Y.-A., Lee J.S., Park J.H., Kim Y.H. (2022). Patterns of physical activity and sedentary behavior and their associated factors among nondisabled stroke survivors. Maturitas.

[55] Gordon N.F., Gulanick M., Costa F., Fletcher G., Franklin B.A., Roth E.J., Shephard T. (2004). Physical activity and exercise recommendations for stroke survivors: An American Heart Association scientific statement from the Council on Clinical Cardiology, Subcommittee on Exercise, Cardiac Rehabilitation, and Prevention; the Council on Cardiovascular Nursing; the Council on Nutrition, Physical Activity, and Metabolism; and the Stroke Council. Circulation.

[56] Wang C., Redgrave J., Shafizadeh M., Majid A., Kilner K., Ali A.N. (2019). Aerobic exercise interventions reduce blood pressure in patients after stroke or transient ischaemic attack: A systematic review and meta-analysis. Br. J. Sports Med..

[57] Chad K.E., Reeder B.A., Harrison E.L., Ashworth N.L., Sheppard S.M., Schultz S.L., Bruner B.G., Fisher K.L., Lawson J.A. (2005). Profile of physical activity levels in community-dwelling older adults. Med. Sci. Sports Exerc..

[58] Juarbe T., Turok X.P., Pérez-Stable E.J. (2002). Perceived benefits and barriers to physical activity among older Latina women. West. J. Nurs. Res..

[59] Salazar M.K. (1990). Interviewer bias: How it affects survey research. Aaohn J..

[60] Marsden D.L., Dunn A., Callister R., McElduff P., Levi C.R., Spratt N.J. (2016). A home-and community-based physical activity program can improve the cardiorespiratory fitness and walking capacity of stroke survivors. J. Stroke Cerebrovasc. Dis..

[61] Lahtio H., Rintala A., Immonen J., Sjögren T. (2022). The Effectiveness of Physical Activity-Promoting Web-and Mobile-Based Distance Weight Loss Interventions on Body Composition in Rehabilitation Settings: Systematic Review, Meta-analysis, and Meta-Regression Analysis. J. Med. Internet Res..

[62] Lear S.A., Hu W., Rangarajan S., Gasevic D., Leong D., Iqbal R., Casanova A., Swaminathan S., Anjana R.M., Kumar R. (2017). The effect of physical activity on mortality and cardiovascular disease in 130 000 people from 17 high-income, middle-income, and low-income countries: The PURE study. Lancet.

