# Supplementary material for: Physical Activity Level, Barriers, and Facilitators for Exercise Engagement for Chronic Community-Dwelling Stroke Survivors in Low-Income Settings: A Cross-Sectional Study in Benin
Source: Int J Environ Res Public Health. 2023 Jan 18;20(3):1784. doi: 10.3390/ijerph20031784 (PMC9914131; doi:10.3390/ijerph20031784)
Supplement: Supplementary file 1 [file ijerph-20-01784-s001.zip › ijerph-2067378-supplementary.pdf]

International Journal of
Environmental Research and Public Health

MDPI

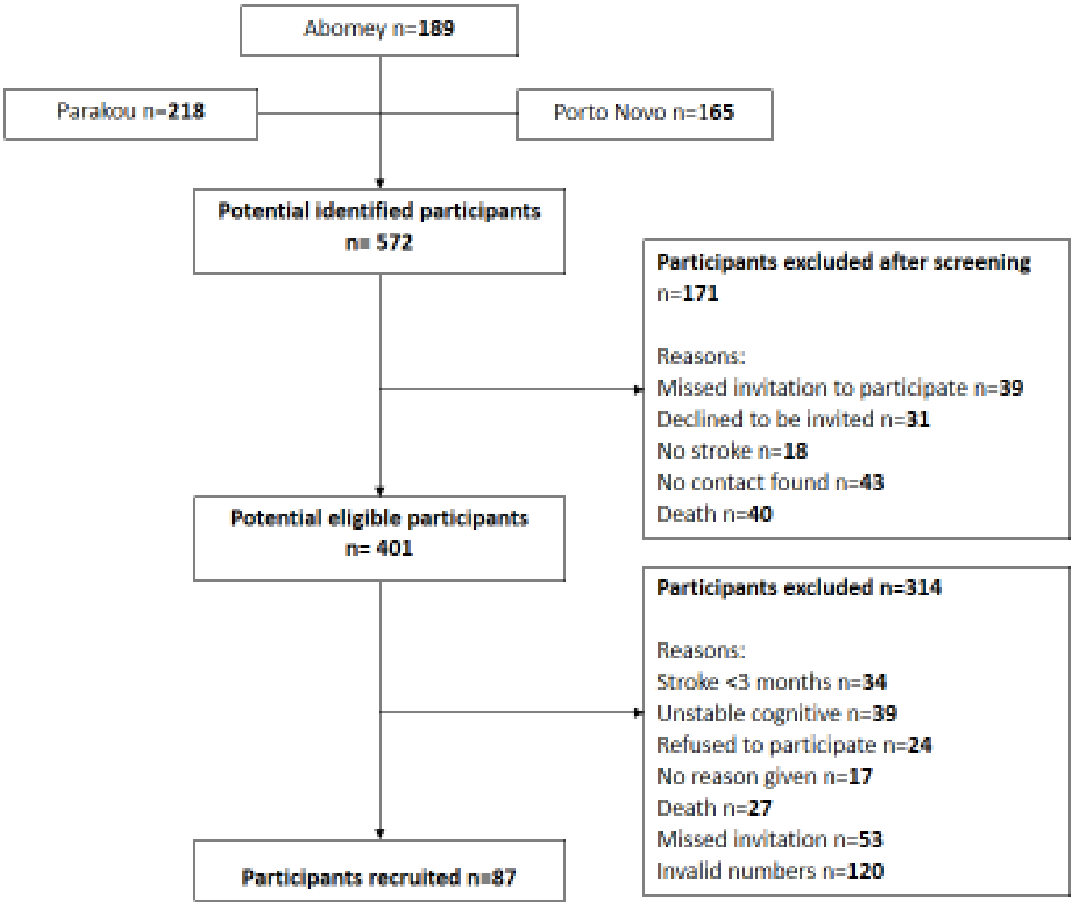

Supplementary Figure S1: Flow chart for participants' selection

Supplementary Table S1. Participant's PA level per domains and category

PA Domains	N	Vigorous PA (%)	Moderate PA (%)	Walking PA (%)	Moderate indoor PA (%)
Work	21	21.9	55.3	22.8	NA
Active Transportation	62	0.0	8.0	92.0	NA
Domestic Domain	35	3.5	56.4	0.0	40.1
Leisure Domain	41	23.9	13.2	62.9	NA

Supplementary Table S2: Total of time (minutes) and day per dominate category

PA Domains	N	Total min	Total day	Mean min	Mean day
**Work (Moderate PA)**	13	1165	65	89.6	5.0
**Active Transportation (Walking)**	61	2340	295	38.4	4.8
**Domestic and gardening (Moderate yard chores)**	15	2190	87	146.0	5.8
**Leisure (Walking)**	27	1615	145	59.8	5.4
